# HDAC3 regulates the diurnal rhythms of claudin expression and intestinal permeability

**DOI:** 10.3389/freae.2024.1496999

**Published:** 2024-11-27

**Authors:** Hunter Christopher, Jianglin Zhang, Sarah Olanrewaju Oladejo, Samskrathi Aravinda Sharma, Zheng Kuang

**Affiliations:** Department of Biological Sciences, Carnegie Mellon University, Pittsburgh, PA, United States

**Keywords:** histone deacetylation, claudin, tight junctions, HDAC3, circadian rhythms, intestinal permeability, RNA-sequencing data analysis

## Abstract

Circadian rhythms play an essential role in the regulation of intestinal absorption and barrier function. Tight junctions, including claudins, are fundamental components of the intestinal epithelial barrier. However, the regulatory mechanisms governing their diurnal expression remain poorly understood. Furthermore, the impact of circadian rhythms on intestinal permeability through claudin modulation has yet to be fully explored. Here we investigated the expression and the diurnal rhythms of claudin transcripts in the intestinal epithelium. We identified histone deacetylase 3 (HDAC3) as an epigenetic regulator that represses claudin expression and drives the diurnal rhythms via histone deacetylation. Loss of HDAC3 leads to increased intestinal permeability and dampened its diurnal rhythm. We further revealed that HDAC3 affects the basolateral localization of claudin-3. Together, our findings give insights into epigenetic modification in regulating tight junction and its diurnal rhythms, providing targets for therapeutic mediations in gastrointestinal disorders.

## 1 Introduction

Circadian rhythms orchestrate cellular functions and synchronized physiological processes with the 24-hour day-night light cycle. Circadian rhythmicity is relevant to a multitude of biological systems. This includes the intestine which is essential to immune system protection, nutrient absorption, and barrier function (Rescigno, 2011). Intestinal epithelial cells rely on tight junctions to sustain barrier integrity, program nutrient absorption and restrict the passage of undesirable substances through the gut lining (Berkes et al., 2003). Many tight junction proteins such as claudins are included in the group of tight junction components, most of which are the contributing factors of paracellular permeability (Günzel and Yu, 2013).

Claudins are generally categorized into two groups: pore-forming claudins, which manage selective channels for small ions and molecules, and barrier-forming claudins, which control paracellular flux by tightening the junctions (Günzel and Yu, 2013). Studies suggest that expression and capacity of claudins are contingent on circadian regulation (Oh-oka et al., 2014). Therefore, disrupting the rhythm of claudins may compromise the intestinal barrier, affect absorption and promote gastrointestinal disorders. However, the diurnal rhythm of claudins and the regulatory mechanisms remain to be determined.

In this study, we explored the expression level and the diurnal rhythm of claudins in the intestinal epithelial cells (IECs). We identified histone deacetylase 3 (HDAC3) as a key epigenetic regulator in programming the diurnal rhythm of claudin expression. HDAC3 interacts with the core circadian clock to affect chromatin structure and gene expression in a time-dependent manner (Shi et al., 2016). Chromatin immunoprecipitation followed by sequencing (ChIP-seq) revealed that HDAC3 represses claudin expression through timely deacetylation of histones across a day-night cycle. This HDAC3-dependent rhythm of claudin expression was correlated with an oscillation of intestinal barrier permeability. We further showed that the cellular localization of claudin-3 was also disrupted in *Hdac3*^*ΔIEC*^ mice. These findings collectively reveal the importance of circadian regulation on intestinal permeability dynamics and suggest the implications for intestinal homeostasis.

## 2 Materials and methods

### 2.1 Mice

C57BL/6 *wildtype* mice, *Hdac3*^*fl/fl*^ and *Hdac3*^*ΔIEC*^ mice were maintained and bred in the SPF barrier at Carnegie Mellon University. *Hdac3*^*ΔIEC*^ mice were generated by crossing a *Hdac3*^*fl/fl*^ mouse and a mouse expressing Cre recombinase under the control of the IEC-specific Villin promoter. All mice were fed *ad libitum* and maintained under a 12-hour light and 12-hour dark cycle.

### 2.2 RNA-sequencing (RNA-seq) data analysis

RNA-seq data were downloaded from Gene Expression Omnibus (GEO) repository with accession number GSE100339 and GSE134303 (Wang et al., 2017; Kuang et al., 2019) and analyzed as previously described (Stappenbeck, Hooper, and Gordon, 2002). In brief, RNA-seq data were mapped against the mm10 genome using TopHat (Trapnell et al., 2012) and fragments per kilobase of transcript per million mapped reads (FPKMs) were generated using Cuffdiff (Trapnell et al., 2012) with default parameters. Circadian oscillations were analyzed by MetaCycle (Wu et al., 2016) with default parameters.

### 2.3 JTK_cycle analysis

Mouse Genome Informatics (MGI) and GEO database annotations were searched to identify entries with ‘tight junction’ in the description. Gene names from the retrieved datasets were cross-referenced with fold-change values obtained from JTK_cycle analysis. Data was normalized to ensure consistency across samples.

### 2.4 Quantitative real-time PCR

Mouse intestine was washed with ice-cold 1x PBS and IECs were extracted using 10 mM EDTA as previously described (Kuang et al., 2019). Total RNA of IECs was extracted using TRI reagent (Sigma, T9424) to synthesize cDNA using Revertaid RT Reverse Transcription kit (Thermo Scientific K1691). qRT-PCR was performed using the Low ROX Forget-Me-Not-EvaGreen qPCR Master Mix (Biotium 31045) on a QuantStudio 5 Real-Time PCR Instrument. The primers used in this study are detailed in Supplementary Table S1. The expression levels of claudin transcripts were normalized to that of Gapdh transcripts.

### 2.5 Chromatin immunoprecipitation sequencing (ChIP-seq)

ChIP-seq data were downloaded from GEO repository with accession number GSE134303 (Kuang et al., 2019). Sequence reads were mapped using BowTie2 (Langmead and Salzberg, 2012). Signals were normalized by the total numbers of aligned reads and visualized using CisGenome Browser (Ji et al., 2008). Peak intensities were quantified in CisGenome Browser and visualized by a heatmap in R.

### 2.6 Western blot analysis

IECs were lysed in the NP-40 lysis buffer (50 mM Tris-HCl, 150 mM NaCl, 1% NP-40, 5 mM EDTA) with protease inhibitor (Roche, 5892791001), phosphatase inhibitor (Thermo Scientific, PIA32957), 1 mM phenylmethylsulfonyl fluoride (Thermo Scientific, PI36978), 1 mM DTT (Thermo Scientific, FERR0861) and 20 mM β-glycerophosphate (Thermo Scientific, 35675). Lysates were prepared through a freeze-thaw cycle three times. Protein concentration was measured by a Pierce BCA kit (Thermo Scientific, PI23227). Equal amounts of protein samples were normalized by concentration. Protein lysates were separated by SDS-PAGE and analyzed by immunoblotting with AZURE biosystem 300. The primary antibodies in this study used were: anti-Claudin-3 (Thermo Scientific, 34–1700) and anti-beta Actin (Cell Signaling, 4970s).

### 2.7 Immunohistochemistry staining

Mouse intestinal tissues were fixed in 4% formaldehyde in 1x PBS overnight at 4°C, and dehydrated in 30% sucrose in 1 × PBS overnight at 4 C. Fixed tissues were embedded in OCT compound (Fisher Scientific, 23730571). Frozen blocks were cut into 7 μm sections and mounted to Superfrost Plus Micro Slides (Fisher Scientific, 1255015). Sections were fixed with 4% paraformaldehyde and washed with 1x PBS three times. Sections were thereafter incubated with a blocking solution (5% BSA, 0.3% Triton X-100) for 1 h, and incubated with anti-Claudin-3 antibody overnight at 4 C in a humidified chamber. Slides were washed with a wash buffer and incubated with secondary antibody for 1 h at room temperature. The slides were washed and mounted with DAPI Fluoromount-G (Southern Biotechnology 0100–20). Images were captured with a fluorescence microscope (Keyence, BZ-X800).

### 2.8 *In vivo* permeability assay

Permeability assay was performed as previously described (Wang et al., 2001; Sigthorsson et al., 2002). Briefly, mice were gavaged with 190 μL 1x PBS/7% DMSO or 1.5 mg/mL indomethacin (Sigma, I7378). After 1 h, mice were gavaged with 190 μL 80 mg/mL FITC-Dextran. After 4 h, serum was collected to detect FITC-dextran levels using a fluorescence microplate reader (Tecan Spark).

## 3 Results

### 3.1 Diurnal expression of claudin genes in mouse intestinal epithelial cells

**FIGURE 1:**
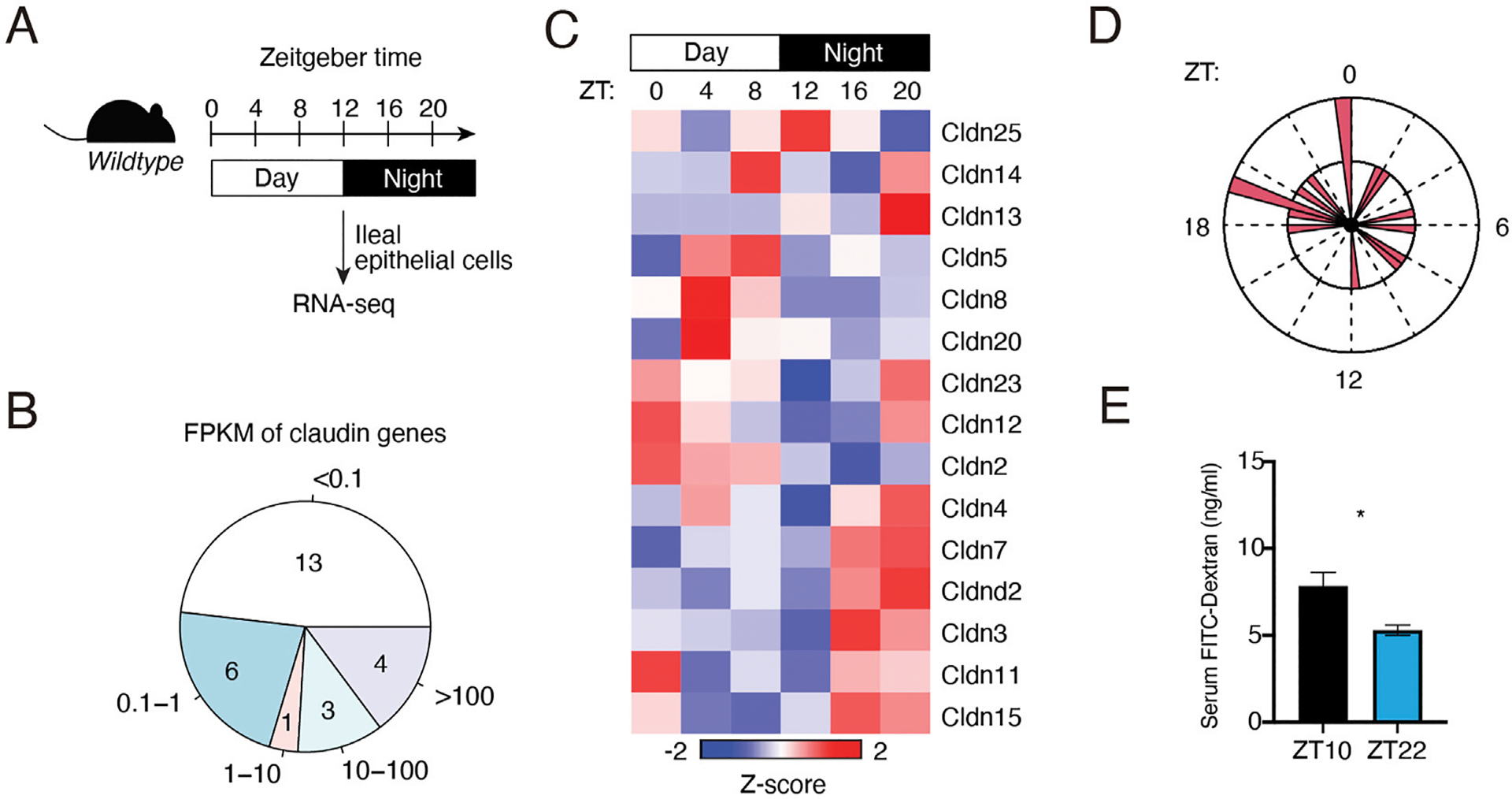
Expression of claudin genes in the intestinal epithelium across a day-night cycle. **(A)** Schematic diagram summarizing the diurnal time points for RNA-seq analysis. **(B)** Expression levels of epithelial claudin genes by RNA-seq. FPKM: fragments per kilobase of transcript per million mapped reads. **(C)** A heat map showing the diurnal expression pattern of claudin genes with FPKM > 0.1. **(D)** A polar chart showing the phase distribution of claudin genes in **(C)**. **(E)** Concentration of serum FITC-dextran of wildtype mice at ZT10 and ZT22. *p < 0.05 by two-tailed *t*-test. Bar chart displays mean ± SEM for five biological replicates (ZT10) and four biological replicates (ZT22). ZT, Zeitgeber time.

To determine the diurnal rhythm of claudin expression in the intestinal epithelium, we analyzed our previous RNA-seq data from *wildtype* mouse IECs across a day-night cycle (Figure 1A). We first examined the expression level of claudin genes based on FPKMs. We found that eight claudin genes had FPKM larger than 1, ordered from high to low as *Cldn7*, *Cldn3*, *Cldn15*, *Cldn4*, *Cldn2*, *Cldn23*, *Cldn25* and *Cldn12* (Figure 1B; Supplementary Figure S1). Another seven claudin genes had FPKM between 0.1 and 1, including *Cldn5*, *Cldn20*, *Cldn8*, *Cldnd2*, *Cldn11*, *Cldn14* and *Cldn13*. This list of claudin genes is consistent with previous studies that these claudin genes are known to be expressed in the intestine (Garcia-Hernandez et al., 2017). Therefore, we focused on the claudin genes with FPKM >0.1 in the following analysis.

Using hierarchical clustering and heatmap analysis, we found that claudin genes were dynamically expressed across a day-night cycle in IECs (Figure 1C). Most of them showed the lowest expression at zeitgeber time (ZT) 12, the beginning of the night. The expression was increased at the end of the nighttime and beginning of the daytime. This was consistent with the circadian phase analysis as shown in Figure 1D. Because claudins play a key role in tight junction integrity and intestinal permeability, we examined the intestinal permeability in mice at dusk (ZT10) and dawn (ZT22) and found that intestinal permeability was higher at ZT10 than ZT22, which is consistent with the dynamics of intestinal permeability from previous studies (Tuganbaev et al., 2020). Thus, the intestinal permeability was rhythmic across the day-night cycle and several claudin genes were found to be diurnally expressed which could explain the permeability dynamics.

### 3.2 Epithelial HDAC3 represses the expression of claudin genes and regulates the diurnal rhythms

**FIGURE 2:**
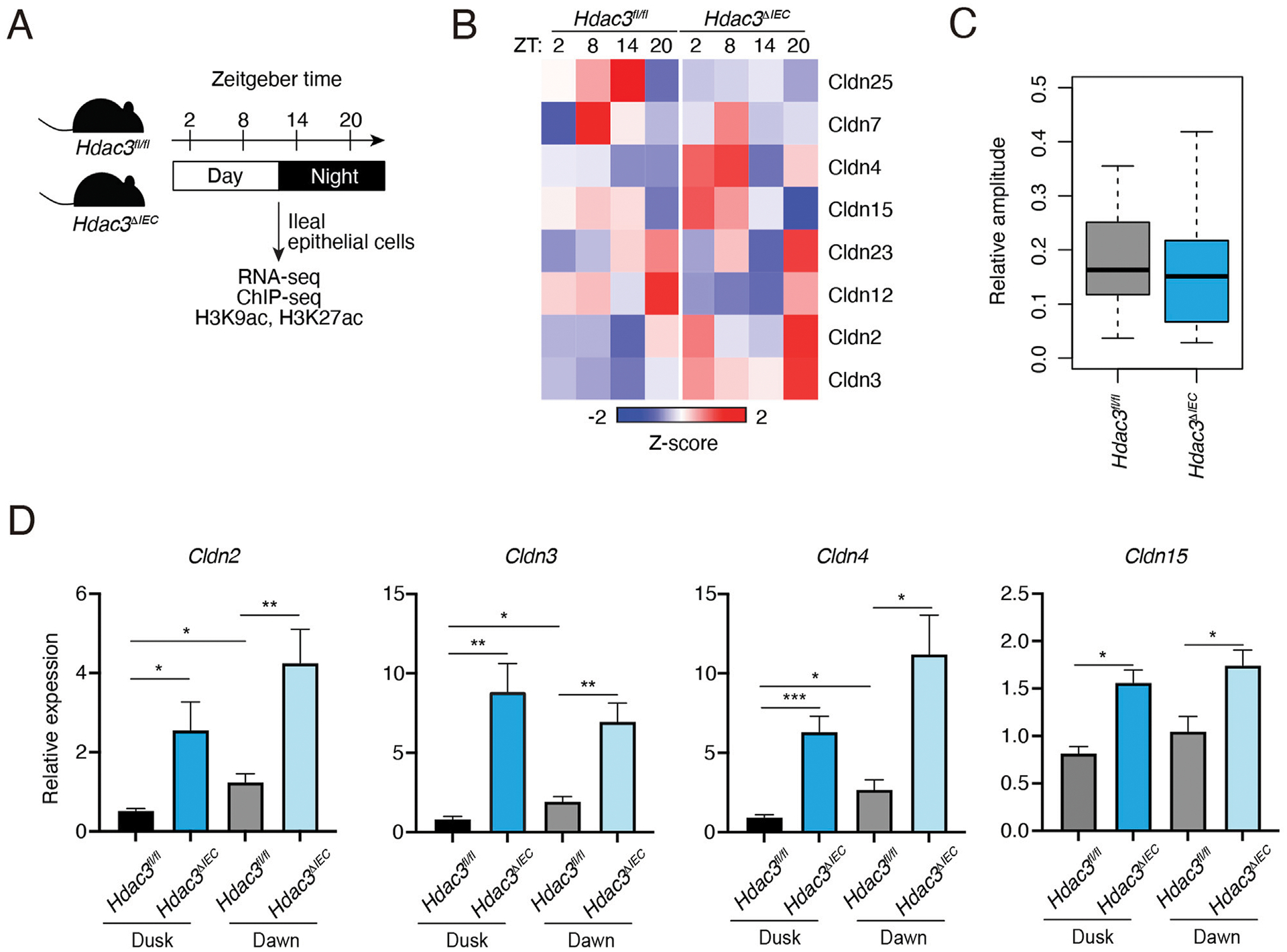
Expression of claudin genes in intestinal epithelial cells in mice lacking HDAC3. **(A)** Schematic diagram summarizing the diurnal time points for RNA-seq and histone acetylation ChIP-seq analysis. **(B)** A heat map showing the diurnal expression pattern of claudin genes with FPKM > 1. **(C)** Relative amplitudes of claudin genes in *Hdac3*^*fl/fl*^ and *Hdac3*^*ΔIEC*^ mice. **(D)** RT-qPCR analysis of some Claudin transcripts in IECs of *Hdac3*^*fl/fl*^ and *Hdac3*^*ΔIEC*^ mice at dusk and dawn. *p < 0.05, **p < 0.01, ***p < 0.001 by two-tailed *t*-test. Bar charts show mean ± SEM for five biological replicates per condition, including both male and female mice. ZT, Zeitgeber time.

Our previous study identified HDAC3 as an important epigenetic regulator of diurnal rhythms in the gut epithelium via histone deacetylation (Kuang et al., 2019). To examine if HDAC3 regulates the diurnal rhythm of claudin gene expression, we analyzed the RNA-seq and histone acetylation ChIP-seq data from previously published datasets in IECs from *Hdac3*^*fl/fl*^ and *Hdac3*^*ΔIEC*^ mice (*Hdac3* is specifically deleted in IECs) across the day-night cycle (Figure 2A). As shown in Figure 2B and Supplementary Figure S2, the expression of many claudin genes was increased in the *Hdac3*^*ΔIEC*^ mice. The relative amplitudes were reduced when HDAC3 was deleted (Figure 2C). Through RNA-seq analysis, we discovered that HDAC3’s regulatory effect also influences other tight junction components (Supplementary Figure S2). We observed changes in the expression of claudin genes as well as other tight junction genes, proposing a more expansive role for HDAC3 in tight junction regulation.

*Cldn2* and *Cldn15* are pore-forming claudins, while *Cldn3* and *Cldn4* are barrier-forming claudins (Günzel and Yu, 2013). We performed RT-qPCR of those four representative claudins in IECs from *Hdac3*^*fl/fl*^ and *Hdac3*^*ΔIEC*^ mice at dusk and dawn to confirm that HDAC3 represses the expression of claudin genes. Consistent with the RNA-seq data, all the four claudins showed higher expression at dawn than the expression at dusk (Figure 2D). When *Hdac3* was deleted, the expression of these claudin genes was increased. We further examined the protein level of one of the claudins, claudin-3 by Western blotting. Claudin-3 protein level was modestly increased in *Hdac3*^*ΔIEC*^ mice, although the upregulation was not as high as the mRNA level (Supplementary Figure S3). Together, these results suggest that HDAC3 can repress the expression of claudin genes and promote the diurnal rhythms in IECs.

### 3.3 Epithelial HDAC3 regulates the intestinal permeability

**FIGURE 3:**
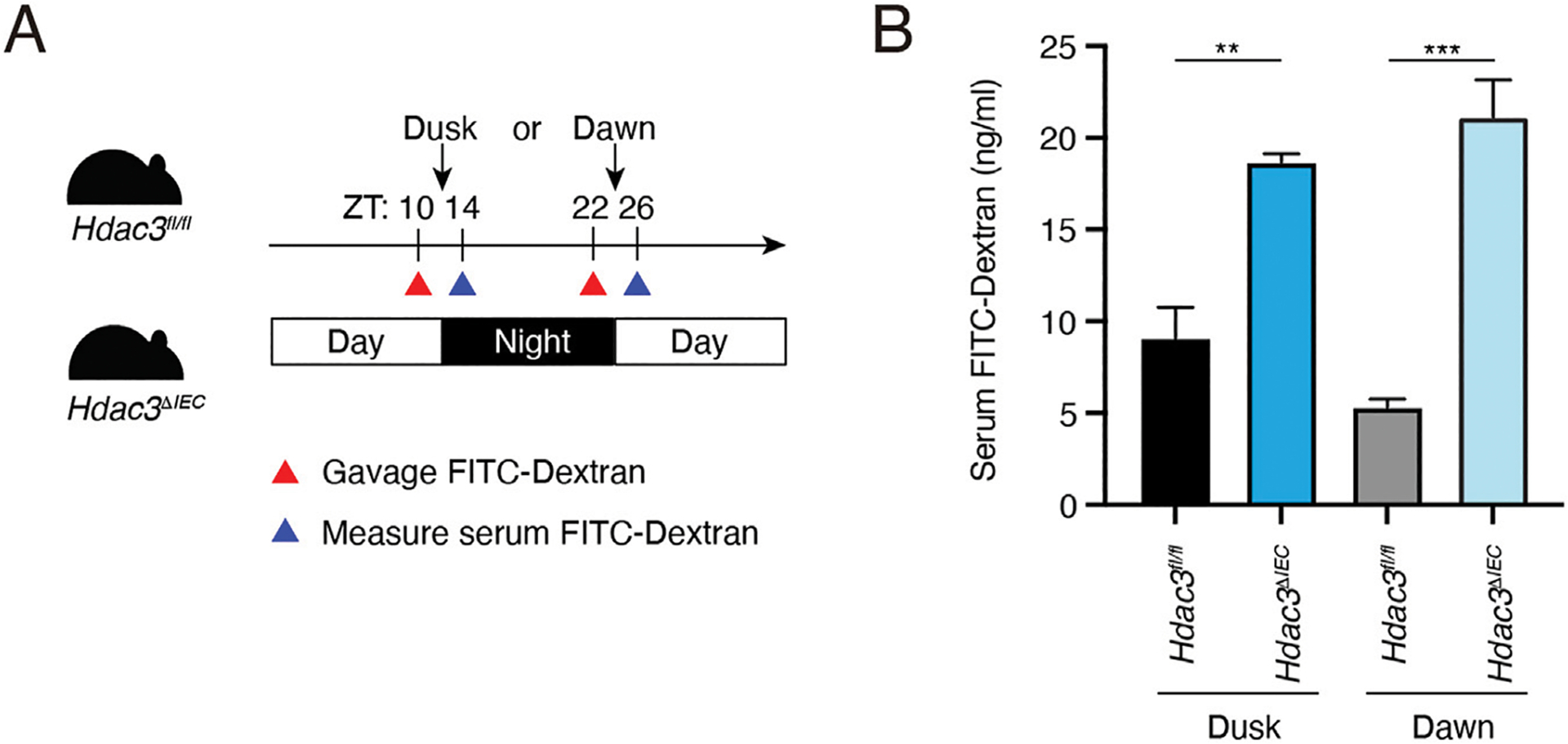
Epithelial HDAC3 regulates the intestinal permeability. **(A)** Schematic diagram of measuring intestinal permeability using FITC-dextran. **(B)** Concentration of serum FITC-dextran of *Hdac3*^*fl/fl*^ and *Hdac3*^*ΔIEC*^ mice at dusk and dawn. **p < 0.01, ***p < 0.001 by two-tailed *t*-test. Bar chart displays mean ± SEM for three to five biological replicates. ZT, Zeitgeber time.

To determine the role of HDAC3 in regulating the diurnal rhythm of intestinal barrier integrity, we measured the intestinal permeability in *Hdac3*^*fl/fl*^ and *Hdac3*^*ΔIEC*^ mice at ZT10 and ZT22 (Figure 3A). At both dusk and dawn, we observed that the intestinal permeability in *Hdac3*^*ΔIEC*^ mice was significantly increased compared to the permeability in *Hdac3*^f*l/fl*^ mice and it did not differ between dusk and dawn (Figure 3B). This result suggests that HDAC3 is required for the intestinal barrier integrity and loss of HDAC3 increases the intestinal permeability and disrupts its diurnal rhythms.

### 3.4 HDAC3 regulates the expression of claudin genes via histone deacetylation

HDAC3 functions canonically as a transcriptional repressor by removing acetylation from histones. Given that claudin gene expression was broadly increased in *Hdac3*^*ΔIEC*^ mice, we explored whether HDAC3 repressed the expression of claudin genes in IECs via histone deacetylation. H3K9ac and H3K27ac are marked as gene promoters and enhancers, and are known to be targeted by HDAC3. These histone acetylation marks open the chromatin structure to enable the binding of activating transcription factors, thus promoting gene expression (Ospelt and Gay, 2013). We previously performed ChIP-seq to examine H3K9ac and H3K27ac in *Hdac3*^*fl/fl*^ and *Hdac3*^*ΔIEC*^ mouse IECs across a day-night cycle (Figure 2A). Thus, we explored this dataset and examined whether HDAC3 regulates the diurnal expression of claudin genes via histone deacetylation.

**FIGURE 4:**
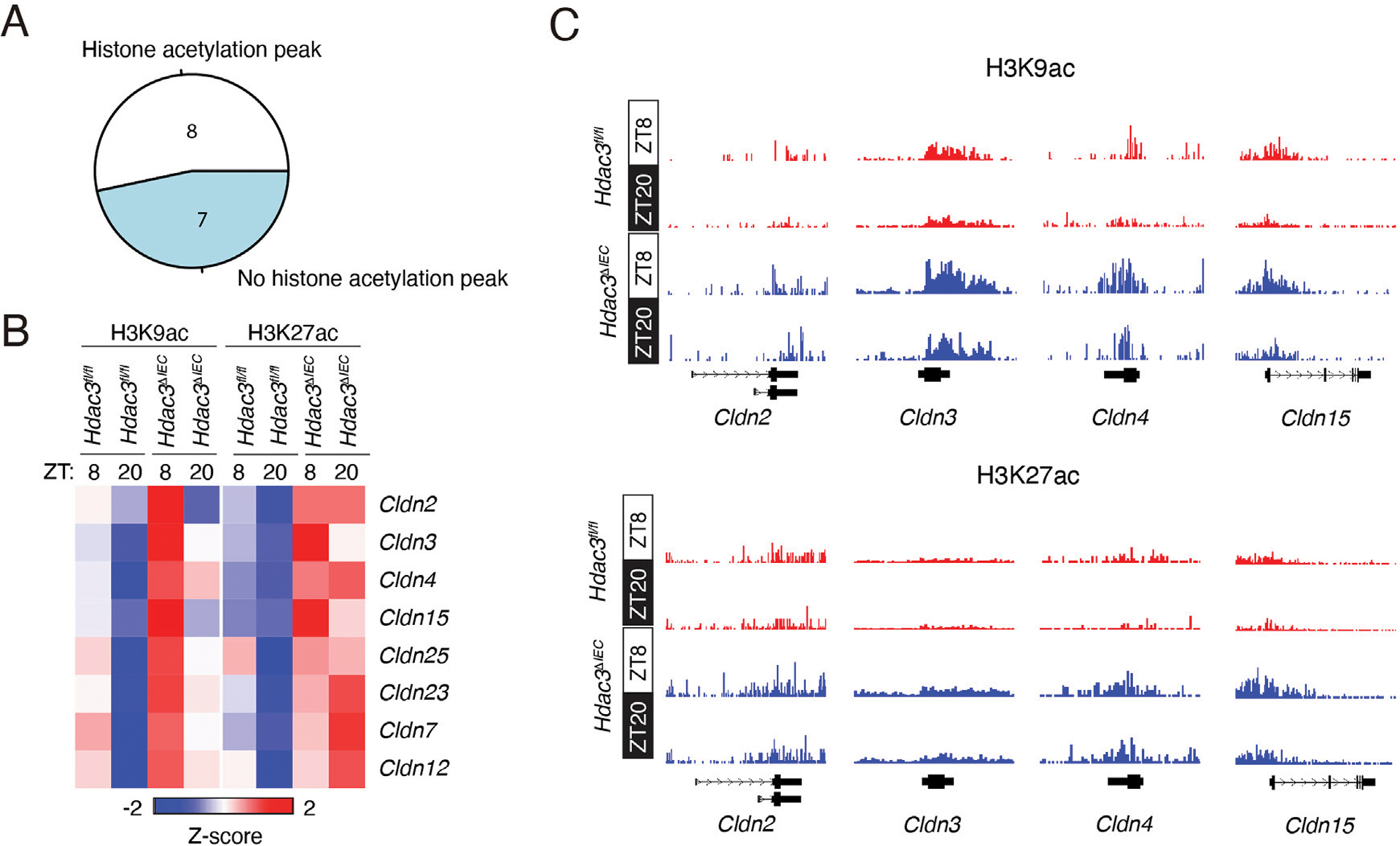
HDAC3 regulates claudin expression via histone deacetylation. **(A)** A pie chart showing the numbers of claudin genes with and without histone acetylation peaks. **(B)** A heat map showing the diurnal signals of H3K9ac and H3K27ac at claudin genes from *Hdac3*^*fl/fl*^ and *Hdac3*^*ΔIEC*^ mice. **(C)** Genome browser view of claudin genes, showing diurnal H3K9ac and H3K27ac signals in IECs.

First, we examined whether H3K9ac and H3K27ac signals are present at claudin genes expressed in the mouse intestine. We found that eight out of the fifteen claudin genes had histone acetylation peaks (Figure 4A). These 8 claudin genes also exhibited FPKMs >1 as shown in Supplementary Figure S1. On the contrary, the other 7 claudin genes did not show strong H3K9ac or H3K27ac peaks at their promoters and the FPKMs were all between 0.1 and 1 (Supplementary Figures S1, S4). Therefore, histone acetylation is enriched at claudin genes that are highly expressed in IECs.

Next, we examined the diurnal pattern of histone acetylation in *Hdac3*^*fl/fl*^ and *Hdac3*^*ΔIEC*^ mouse IECs. Both H3K9ac and H3K27ac signals were synchronized at claudin genes in *Hdac3*^*fl/fl*^ mice and were high at ZT8 and low at ZT20 (Figures 4B, C). However, in *Hdac3*^*ΔIEC*^ mice, H3K9ac and H3K27ac were dramatically increased. The diurnal rhythm of H3K9ac was dampened and the rhythm of H3K27ac was almost abolished. Therefore, these results suggest that HDAC3 represses claudin gene expression by deacetylating histones in a diurnal manner.

### 3.5 HDAC3 regulates basolateral localization of CLDN-3 in intestinal epithelial cells

**FIGURE 5:**
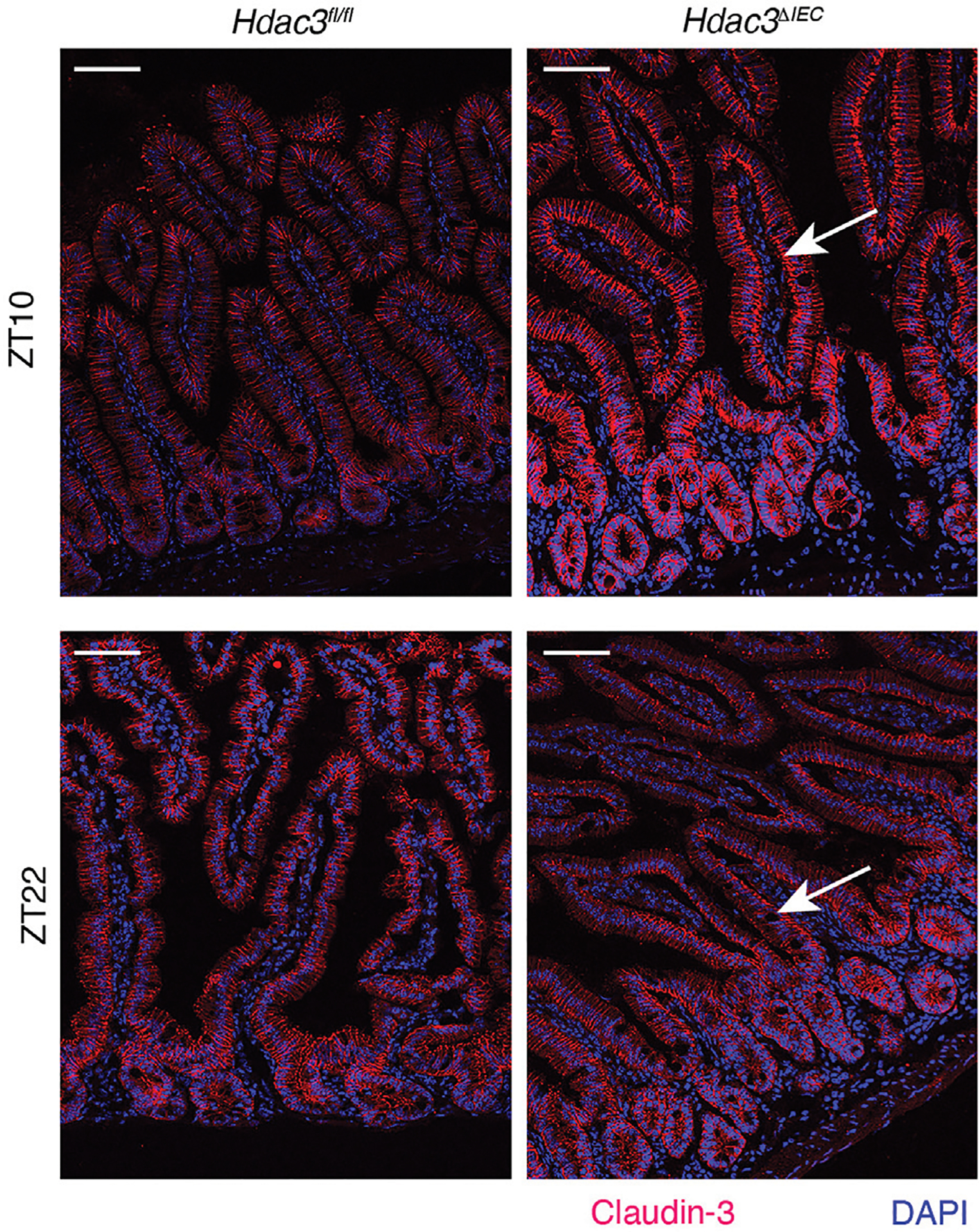
HDAC3 regulates cellular localization of Claudin-3. Immunofluorescence staining of Claudin-3 in small intestinal tissues from *Hdac3*^*fl/fl*^ and *Hdac3*^*ΔIEC*^ mice at ZT10 and ZT22. Scale bar = 40 μm.

Besides the expression, the cellular localization of claudins is also important for their functions in tight junctions. Analyzing the expression and localization pattern of claudins is an important tool for prognostic predictors in many diseases, such as cancer (Resnick et al., 2005; Bhat et al., 2020). To examine the localization of claudin-3, we performed immunofluorescence staining in female mouse intestinal epithelial tissues (Figure 5). In *Hdac3*^*fl/fl*^ mice, claudin-3 is predominantly localized in the lateral areas of the intestinal epithelial cells. Conversely, in *Hdac3*^*ΔIEC*^ mice, claudin-3 shows a marked shift in localization, being enriched in the basolateral areas of the intestinal epithelial cells. We then repeated this experiment with male mouse intestinal tissues (Supplementary Figure S5), where we observed localization that mirrored the female intestine tissues: lateral localization in *Hdac3*^*fl/fl*^ mice and basolateral localization in *Hdac3*^*ΔIEC*^ mice. This altered localization suggests that HDAC3 plays a role in the spatial distribution of claudin-3. In *Hdac3*^*ΔIEC*^ mice, the absence of HDAC3 disrupts this normal lateral localization pattern of claudin-3, leading to the basolateral enrichment. This mislocalization could compromise the epithelial barrier integrity and the function of tight junctions, resulting in increased susceptibility to intestinal disorders and disruption of intestinal homeostasis.

## 4 Discussion

**FIGURE 6:**
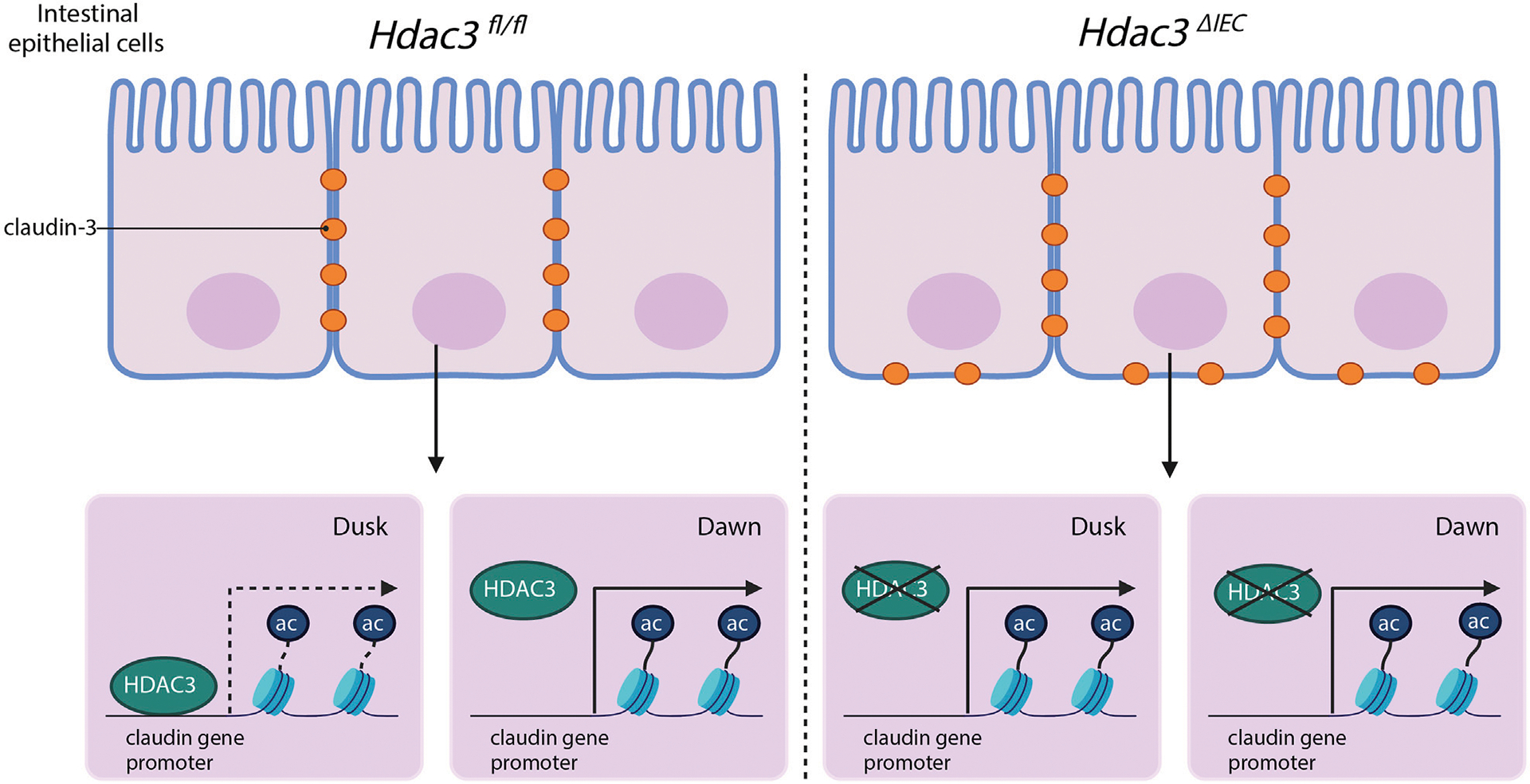
The hypothesized model of HDAC3 regulating the diurnal rhythms in claudin expression and intestinal permeability.

This study advances our understanding of mucosal barrier functions by demonstrating that claudins, essential components of tight junctions, have diurnal rhythmicity in their expression (Figure 6). Tight junction proteins are not simply structural elements; they coordinate with each other (Lal-Nag and Morin, 2009). We have shown that the expression of claudins in IECs is regulated in a time-dependent manner. We have also identified an epigenetic pathway that controls the diurnal expression of claudins, involving histone acetylation and HDAC3 specifically. These findings open a new avenue for research on how disruption of circadian rhythms could contribute to intestinal disorders and offer potential targets for therapeutic intervention.

Our findings reveal the probable impact of circadian regulation on intestinal permeability, potentially through the control of rhythmic expression of claudins. The gut luminal environment is rhythmic, exemplified by oscillations of food availability and microbial load (Dantas Machado et al., 2022; Wang et al., 2022). This rhythmic claudin expression and permeability could play a part in the optimization of nutrient absorption while maintaining barrier integrity. As stated previously, claudins are grouped into two categories, pore-forming and barrier-forming. While our findings reveal that both types of claudins exhibit diurnal rhythms of expression, how they interact and collectively program the rhythm in intestinal permeability remains to be determined.

While this study produces new insights into the circadian regulation of claudins and the role of HDAC3, there are limitations. Claudins act within a complex network of interactions, so other factors or tight junction proteins may contribute to the intestinal barrier’s circadian modulation. While we showed that other tight junction components were also increased when HDAC3 was disrupted (Supplementary Figure S2C, D), the exact mechanism by which HDAC3 effectuates these outcomes remains to be fully resolved. Further investigation is needed on whether HDAC3 directly targets specific claudin genes or influences their expression through a broader chromatin remodeling activity that operates on multiple claudin or tight junction components. Additionally, we showed that HDAC3 also affects the basolateral localization of claudin-3, which is unexpected given HDAC3’s canonical roles in histone deacetylation and transcriptional repression. How might HDAC3 regulate the localization of claudin-3? HDAC3 is recognized for removing acetyl groups from histones and non-histone proteins, which ultimately influences the activity of said protein. There is a possibility that HDAC3 could actively regulate the acetylation state of claudin-3 if it is a substrate of HDAC3. Future experiments can assess acetylation levels of claudin-3 in models with a functioning HDAC3 versus those with HDAC3 disrupted. Significance in acetylation levels between the two groups would suggest that HDAC3 impacts the acetylation of claudin-3. Acetylation is a modification that can substantially influence the function of proteins and by extension localization. With respect to a protein that is critical for tight junction integrity like claudin-3, acetylation might impact its capability to bind to other tight junction complexes. Future research could use a claudin-3 mutant which cannot be acetylated and compare it to a wild-type claudin-3 to assess its localization. Addressing these questions may provide important new insights into claudin biology and intestinal barrier regulation.

Furthermore, the broader implications of our findings for health and disease are speculative. Future research could focus on bridging the gap by examining the relationship between claudin expression, circadian disruption and potential intestinal disorders in clinical settings. Considering conditions where excessive permeability occurs, such as a leaky gut, understanding the process by which HDAC3 selectively modulates pore-forming versus barrier-forming claudins could help significantly. Treatment that adjusts the relative abundance of functional claudins based on clinical needs could improve symptoms.

Disturbance of the biological clock triggers inflammation (Giebfried and Lorentz, 2023). The findings of this paper can potentially help night-shift workers and people who are jet lagged who experience severe Inflammatory Bowel Disease (IBD) symptoms to restore their circadian rhythms. Disorders surrounding epigenetic dysregulation would benefit from treatments focusing on specific epigenetic pathways, particularly those involved in circadian regulation. Researchers have identified HDAC3 activators that promote gut repair via phytate metabolism (Wu et al., 2020). Similar mechanisms may be repurposed to target barrier regulation through tight junctions. This has the potential to dismantle fluctuations in the gut barrier caused by disrupted circadian management.

Together, this study reveals intricate connections between epigenetic regulation, the circadian rhythm and intestinal barrier function. Making these connections clear will create a passage for possible therapeutic interventions in disorders related to circadian disruption and gut barrier dysfunction.

## Supplementary Material

Table 1

Figure S4

Figure S5

Figure S3

Figure S2

Figure S1

## Data Availability

The data presented in the study are deposited in the Gene Expression Omnibus repository, accession numbers GSE100339 and GSE134303.
