# Supplementary material for: HDAC3 regulates the diurnal rhythms of claudin expression and intestinal permeability
Source: Front Epigenet Epigenom. 2024 Nov 27;2:1496999. doi: 10.3389/freae.2024.1496999 (PMC12320956; doi:10.3389/freae.2024.1496999)
Supplement: Supplementary file 2 [file DataSheet2.PDF]

# Figure S2

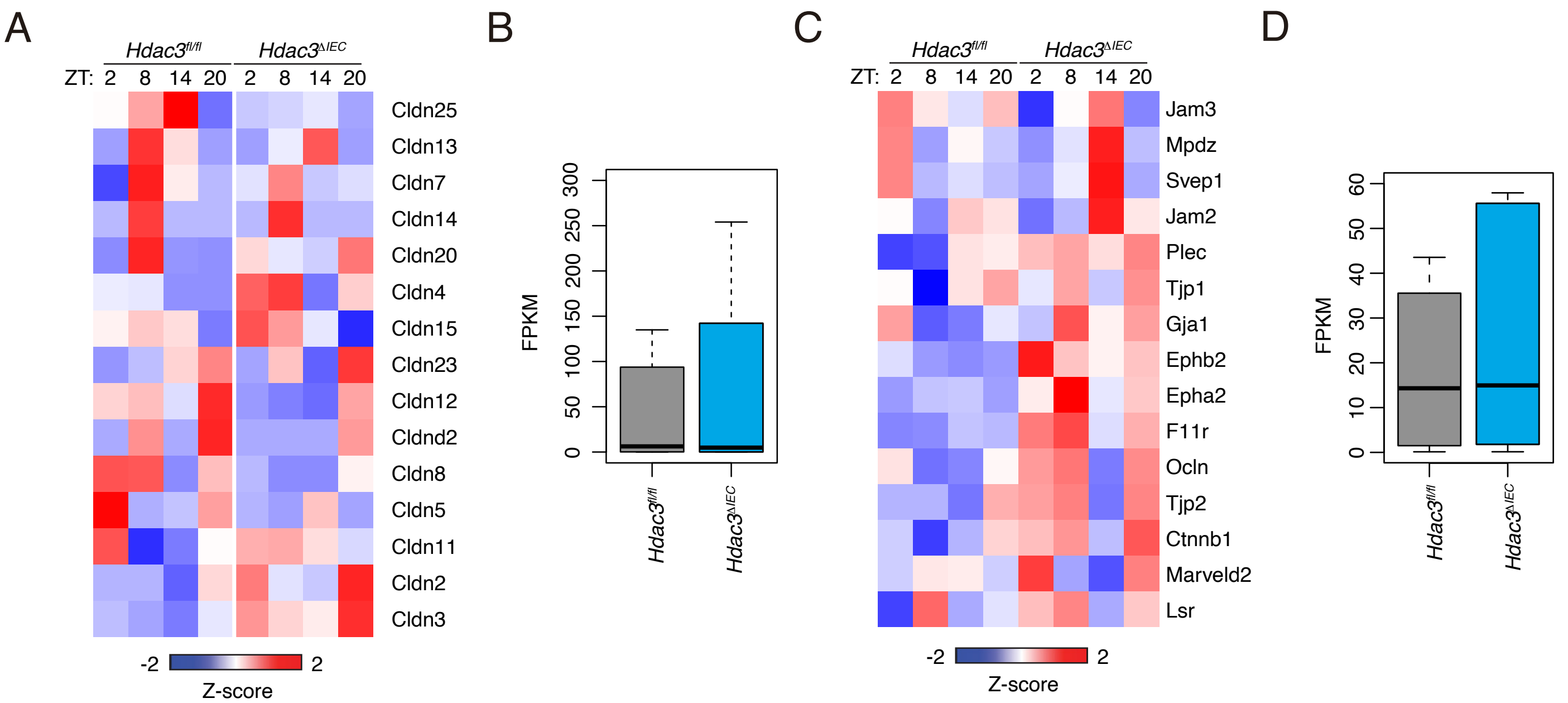


**Supplementary Figure S2. Epithelial HDAC3 regulates claudin gene expression. (A)** A heat map showing the diurnal expression pattern of claudin genes in IECs of *Hdac3$^{fl/fl}$* and *Hdac3$^{ΔIEC}$* mice. Claudin genes with FPKM > 0.1 were included. **(B)** Average expression of claudin genes in IECs of *Hdac3$^{fl/fl}$* and *Hdac3$^{ΔIEC}$* mice. **(C)** A heat map showing the diurnal expression pattern of other tight junction genes in IECs of *Hdac3$^{fl/fl}$* and *Hdac3$^{ΔIEC}$* mice. **(D)** Average expression of other tight junction genes in IECs of *Hdac3$^{fl/fl}$* and *Hdac3$^{ΔIEC}$* mice.
