# Supplementary material for: HDAC3 regulates the diurnal rhythms of claudin expression and intestinal permeability
Source: Front Epigenet Epigenom. 2024 Nov 27;2:1496999. doi: 10.3389/freae.2024.1496999 (PMC12320956; doi:10.3389/freae.2024.1496999)
Supplement: Supplementary file 3 [file DataSheet3.PDF]

Figure S3

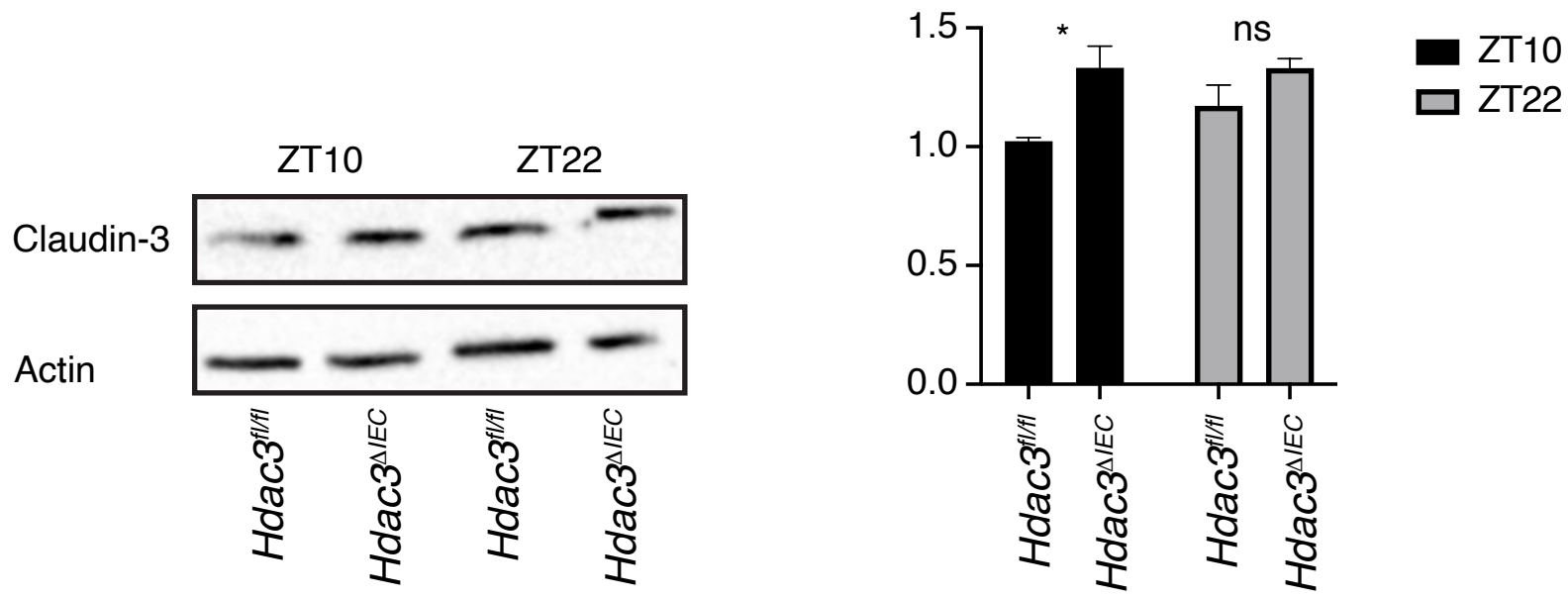

Supplementary Figure S3. Protein level of Claudin 3 in IECs of *Hdac3$^{fl/fl}$* and *Hdac3$^{\Delta IEC}$* mice at ZT10 and ZT22. Bar chart displays mean ± SEM for two to three repeat experiments where IECs from three to five mice were pooled together.
