# Supplementary material for: HDAC3 regulates the diurnal rhythms of claudin expression and intestinal permeability
Source: Front Epigenet Epigenom. 2024 Nov 27;2:1496999. doi: 10.3389/freae.2024.1496999 (PMC12320956; doi:10.3389/freae.2024.1496999)
Supplement: Supplementary file 5 [file DataSheet5.PDF]

Figure S5

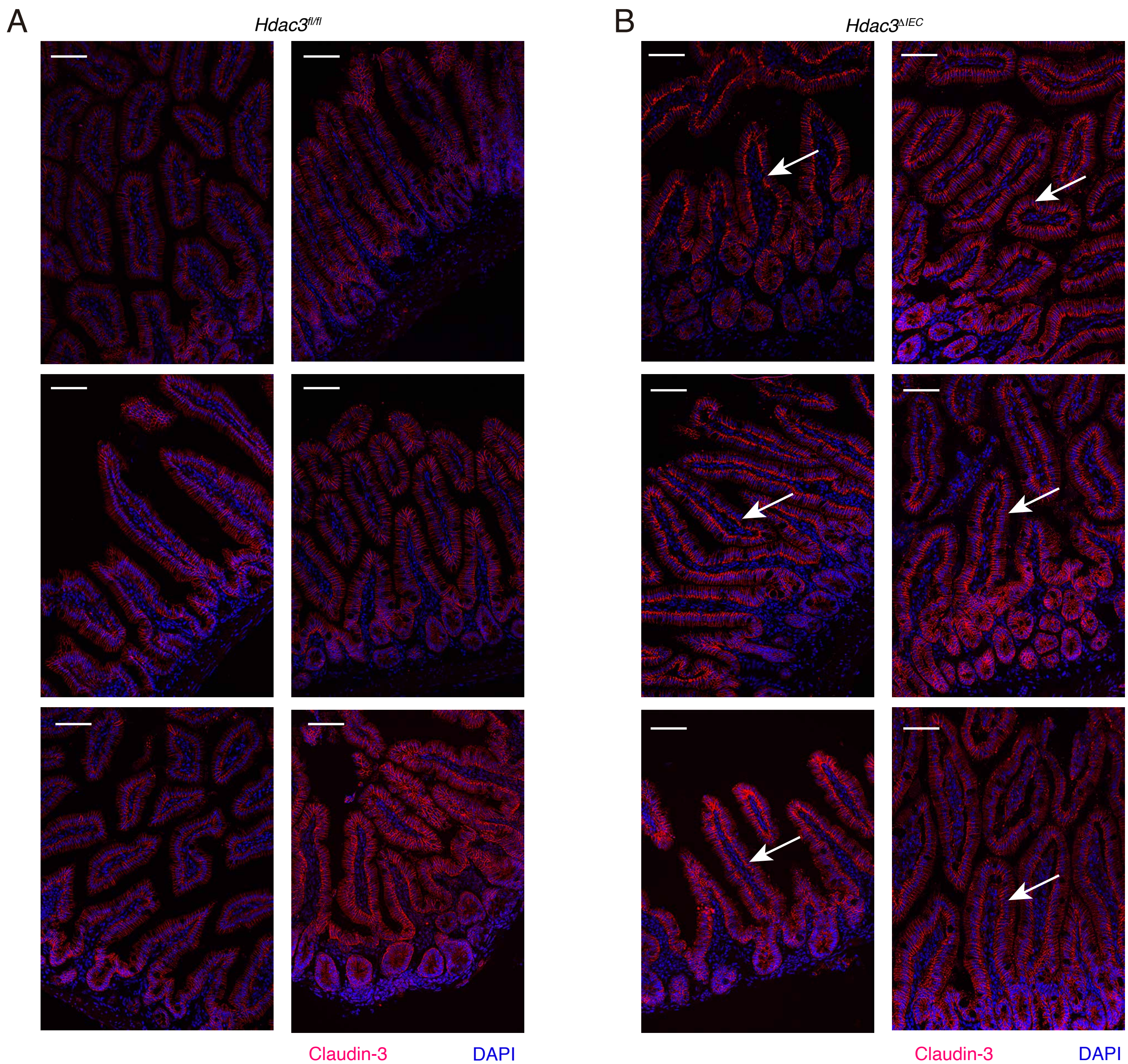

Supplementary Figure S5. Localization of Claudin-3 in male *Hdac3*$^{fl/fl}$ and *Hdac3*$^{\Delta IEC}$ mouse small intestine. Immunofluorescence staining of Claudin-3 in small intestinal tissues from *Hdac3*$^{fl/fl}$ (A) and *Hdac3*$^{\Delta IEC}$ (B) mice. Each image is a representative view of different mice. Scale bar = 40 μm.
