# Supplementary material for: HDAC3 regulates the diurnal rhythms of claudin expression and intestinal permeability
Source: Front Epigenet Epigenom. Author manuscript; Available in PMC 2025 Aug 4. (PMC12320956; doi:10.3389/freae.2024.1496999)
Supplement: Figure S4 [file NIHMS2039487-supplement-Figure_S4.pdf]

Figure S4

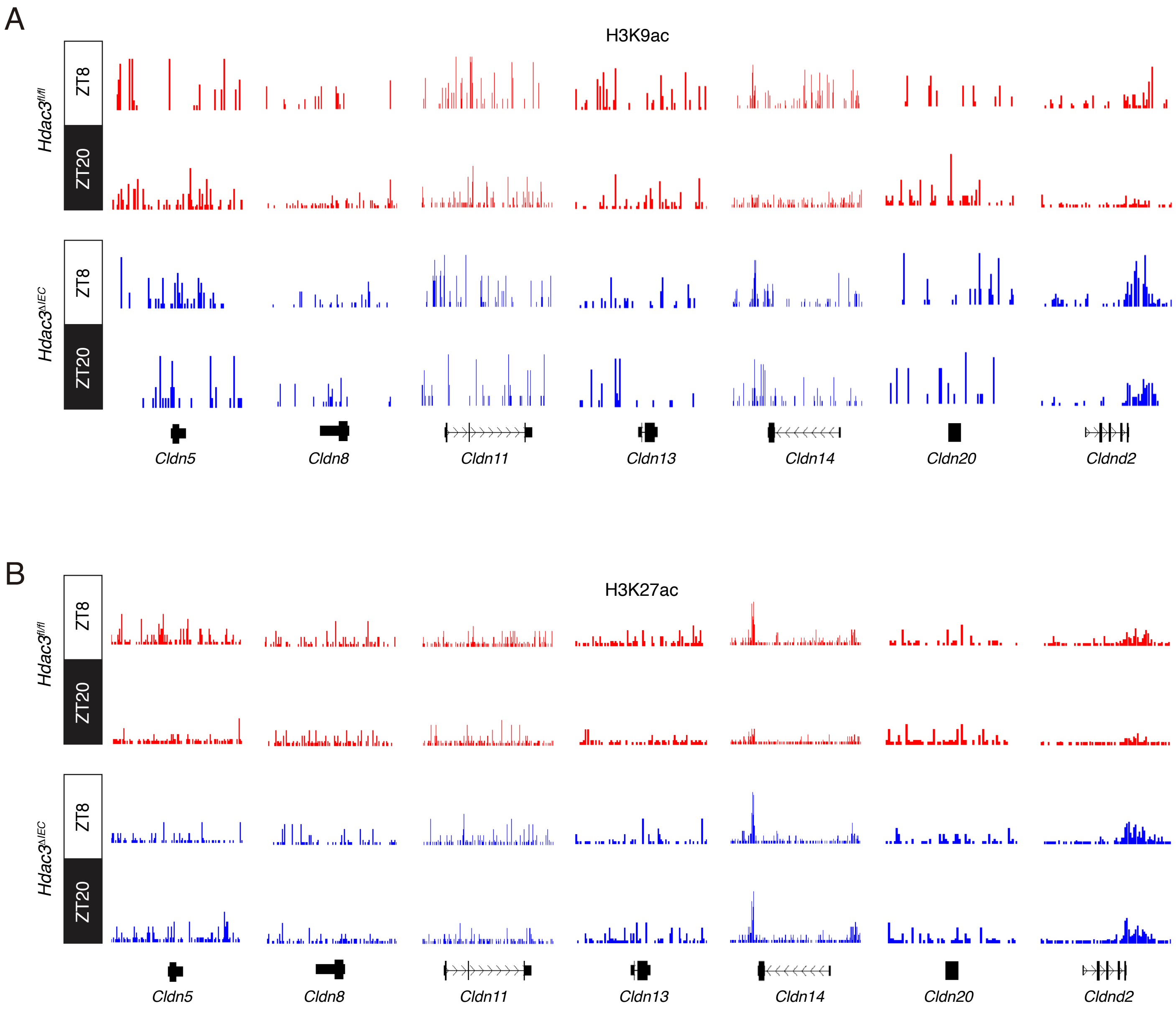

Supplemenatry Figure S4. Genome browser view of claudin genes, showing H3K9ac **(A)** and H3K27ac **(B)** signals in IECs. Claudin genes with FPKM < 1 were included.
