# Supplementary material for: HDAC3 regulates the diurnal rhythms of claudin expression and intestinal permeability
Source: Front Epigenet Epigenom. Author manuscript; Available in PMC 2025 Aug 4. (PMC12320956; doi:10.3389/freae.2024.1496999)
Supplement: Figure S5 [file NIHMS2039487-supplement-Figure_S5.pdf]

Figure S5

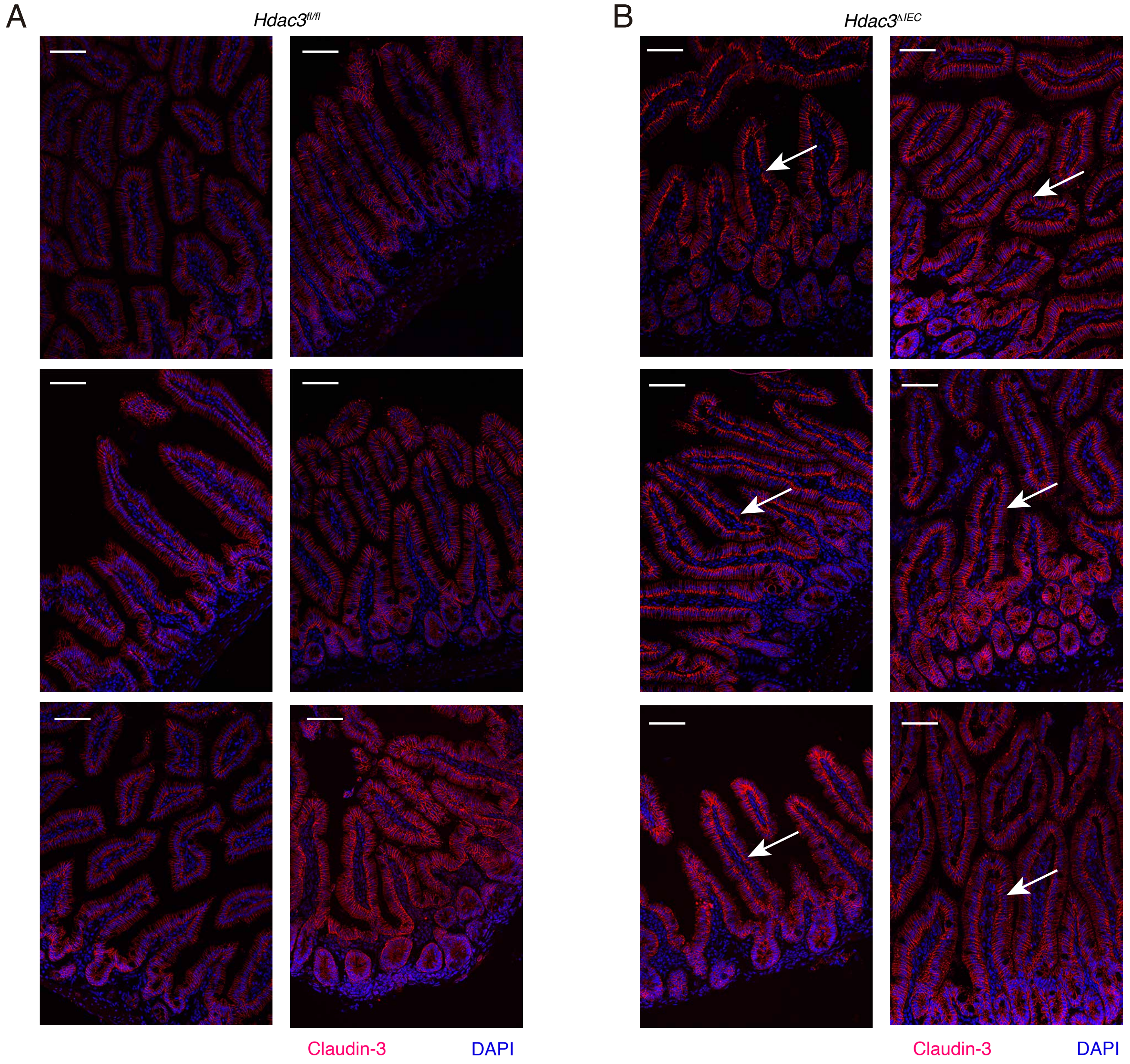

Supplementary Figure S5. Localization of Claudin-3 in male *Hdac3$^{fl/fl}$* and *Hdac3$^{\Delta IEC}$* mouse small intestine. Immunofluorescence staining of Claudin-3 in small intestinal tissues from *Hdac3$^{fl/fl}$* (A) and *Hdac3$^{\Delta IEC}$* (B) mice. Each image is a representative view of different mice. Scale bar = 40 μm.
