# Supplementary material for: HDAC3 regulates the diurnal rhythms of claudin expression and intestinal permeability
Source: Front Epigenet Epigenom. Author manuscript; Available in PMC 2025 Aug 4. (PMC12320956; doi:10.3389/freae.2024.1496999)
Supplement: Figure S3 [file NIHMS2039487-supplement-Figure_S3.pdf]

Figure S3

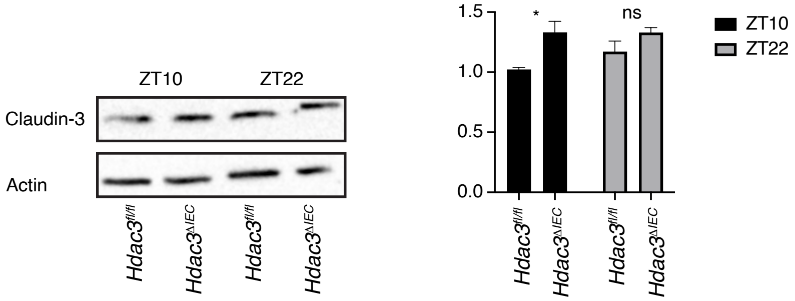

Supplementary Figure S3. Protein level of Claudin 3 in IECs of *Hdac3*$^{fl/fl}$ and *Hdac3*$^{\Delta IEC}$ mice at ZT10 and ZT22. Bar chart displays mean ± SEM for two to three repeat experiments where IECs from three to five mice were pooled together.
