# Supplementary material for: HDAC3 regulates the diurnal rhythms of claudin expression and intestinal permeability
Source: Front Epigenet Epigenom. Author manuscript; Available in PMC 2025 Aug 4. (PMC12320956; doi:10.3389/freae.2024.1496999)
Supplement: Figure S2 [file NIHMS2039487-supplement-Figure_S2.pdf]

Figure S2

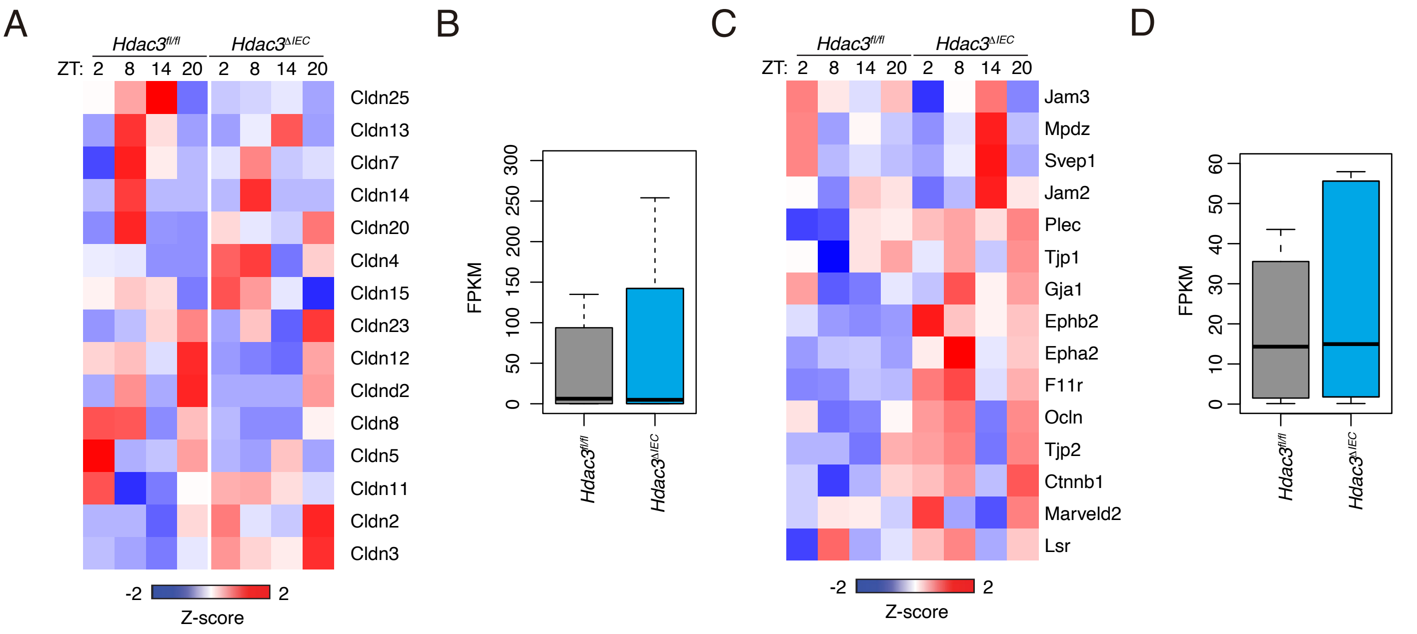

Supplementary Figure S2. Epithelial HDAC3 regulates claudin gene expression. (A) A heat map showing the diurnal expression pattern of claudin genes in IECs of *Hdac3*$^{fl/fl}$ and *Hdac3*$^{\Delta IEC}$ mice. Claudin genes with FPKM > 0.1 were included. **(B)** Average expression of claudin genes in IECs of *Hdac3*$^{fl/fl}$ and *Hdac3*$^{\Delta IEC}$ mice. **(C)** A heat map showing the diurnal expression pattern of other tight junction genes in IECs of *Hdac3*$^{fl/fl}$ and *Hdac3*$^{\Delta IEC}$ mice. **(D)** Average expression of other tight junction genes in IECs of *Hdac3*$^{fl/fl}$ and *Hdac3*$^{\Delta IEC}$ mice.
