# Supplementary material for: HDAC3 regulates the diurnal rhythms of claudin expression and intestinal permeability
Source: Front Epigenet Epigenom. Author manuscript; Available in PMC 2025 Aug 4. (PMC12320956; doi:10.3389/freae.2024.1496999)
Supplement: Figure S1 [file NIHMS2039487-supplement-Figure_S1.pdf]

Figure S1

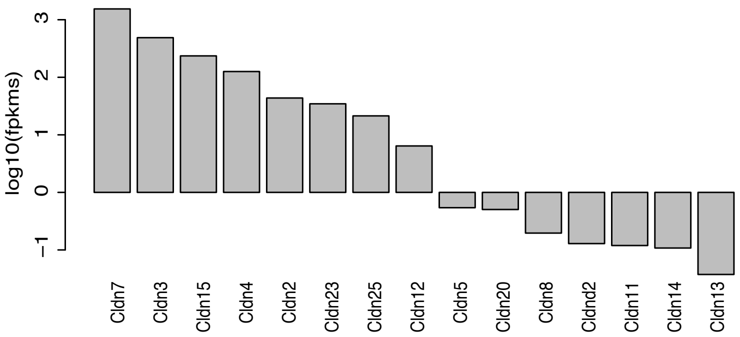

Supplementary Figure S1. Expression levels of claudin genes in the intestinal epithelium by RNA-seq analysis. Expression was measured by FPKM (fragments per kilobase of transcript per million mapped reads), and claudin transcripts were ordered from highest to lowest.
